# Enterovirus D‐68 in children presenting for acute care in the hospital setting

**DOI:** 10.1111/irv.12551

**Published:** 2018-03-23

**Authors:** Timothy J. Savage, Jane Kuypers, Helen Y. Chu, Miranda C. Bradford, Anne Marie Buccat, Xuan Qin, Eileen J. Klein, Keith R. Jerome, Janet A. Englund, Alpana Waghmare

**Affiliations:** ^1^ Seattle Children's Hospital Seattle WA USA; ^2^ University of Washington Seattle WA USA; ^3^ Seattle Children's Research Institute Seattle WA USA; ^4^ Fred Hutchinson Cancer Research Center Seattle WA USA

**Keywords:** enterovirus, enterovirus‐D68, respiratory infection, rhinovirus

## Abstract

**Background:**

Severe respiratory disease associated with enterovirus D68 (EV‐D68) has been reported in hospitalized pediatric patients. Virologic and clinical characteristics of EV‐D68 infections exclusively in patients presenting to a hospital Emergency Department (ED) or urgent care have not been well defined.

**Methods:**

Mid‐nasal swabs from pediatric patients with respiratory symptoms presenting to the ED or urgent care were evaluated using a commercial multiplex PCR platform. Specimens positive for rhinovirus/enterovirus (HRV/EV) were subsequently tested using real‐time reverse‐transcriptase PCR for EV‐D68. The PCR cycle threshold (CT) was used as a viral load proxy. Clinical outcomes were compared between patients with EV‐D68 and patients without EV‐D68 who tested positive for HRV/EV.

**Results:**

From August to December 2014, 511 swabs from patients with HRV/EV were available. EV‐D68 was detected in 170 (33%) HRV/EV‐positive samples. In multivariable models adjusted for age and underlying asthma, patients with EV‐D68 were more likely to require hospitalization for respiratory reasons (odds ratio (OR): 3.11, CI: 1.85‐5.25), require respiratory support (OR: 1.69, CI: 1.09‐2.62), have confirmed/probable lower respiratory tract infection (LRTI; OR: 3.78, CI: 2.03‐7.04), and require continuous albuterol or steroids (OR: 3.91, CI: 2.22‐6.88 and OR: 4.73, CI: 2.65‐8.46, respectively). Higher EV‐D68 viral load was associated with need for respiratory support and LRTI in multivariate models.

**Conclusions:**

Among pediatric patients presenting to the ED or urgent care, EV‐D68 causes more severe disease than non‐EV‐D68 HRV/EV independent of underlying asthma. High viral load was associated with worse clinical outcomes. Rapid and quantitative viral testing may help identify and risk stratify patients.

## 1 INTRODUCTION

Enterovirus D68 (EV‐D68) is a non‐polio enterovirus recently recognized as a significant cause of respiratory disease after decades of sporadic reported cases and small outbreaks.^1^, ^2^ A spike in nationwide pediatric admissions due to respiratory symptoms in summer 2014 prompted increased surveillance that led to the detection of EV‐D68 as the likely responsible pathogen.^3^, ^4^, ^5^, ^6^ Initially, hospitals in the American Midwest reported increased burdens on emergency departments^7^ and described severe respiratory illness caused by EV‐D68 among children admitted to the pediatric intensive care unit.^8^ We identified a concurrent outbreak at Seattle Children's Hospital that led to a significant increase in the number of EV‐D68 cases detected.

Recent studies have added to our knowledge of the burden of disease of EV‐D68 in the inpatient and ICU settings^8^, ^9^, ^10^, ^11^, ^12^, ^13^ and in immunocompromised patients,^14^ but less is known about patients with EV‐D68 presenting to a hospital for acute care in the urgent care or emergency department (ED). Factors associated with inpatient admission in these patients are not well described, and the role of viral load in disease severity has not been investigated systematically. Pediatric patients with asthma are of particular interest as asthma affects nearly 10% of children,^15^ and data regarding EV‐D68 disease severity within this group have been conflicting.^8^, ^10^, ^12^, ^13^, ^16^ Our study aims to define the burden of EV‐D68 in a large cohort of pediatric patients presenting for urgent or emergent care in the ED or urgent care clinic at a regional children's hospital and to compare the clinical presentation, role of comorbidities, and outcomes between patients with EV‐D68 and those with other strains of human rhinovirus/enterovirus (HRV/EV) during an outbreak.

## 2 METHODS

### 2.1 Patients and study setting

Patients presenting to Seattle Children's Hospital ED or urgent care clinic with respiratory symptoms from August to December of 2014 were included in the study. Patients underwent respiratory viral testing as clinically indicated at the time of their evaluation, and all patients with respiratory symptoms who were admitted to the hospital were tested for respiratory viruses for cohorting purposes based on hospital policy. Patients were eligible for inclusion if they were tested in the ED or urgent care or within 24 hours of hospital admission.

### 2.2 Virus identification and quantification

Mid‐nasal turbinate swabs were collected, and tests were resulted within 2 hours of collection using the FilmArray multiplex polymerase chain reaction (PCR) respiratory panel (BioFire, Salt Lake City, UT). This platform does not differentiate between HRV and EVs, and positive tests are reported as “rhinovirus/enterovirus” (HRV/EV). Patient samples testing positive for HRV/EV were subsequently tested at the University of Washington; 200 uL of each sample was extracted and eluted in 200 uL of buffer. The samples were then tested by a real‐time reverse‐transcriptase PCR targeting the EV‐D68 5’ non‐coding region using forward primer GCGTTGGCGGCCTACTC and a previously published reverse primer and FAM‐labeled probe.^17^ The EV‐D68 RT‐PCR assay was performed by adding 10 uL of extracted nucleic acids to AgPath One‐Step RT‐PCR master mix (Thermo Fisher) with final concentrations of 500 nmol/L for each primer and 100 nmol/L of the FAM‐labeled probe in a total reaction volume of 35 uL.

The PCR cycle threshold (CT) was used as a proxy for viral load with lower CT indicating a higher viral load. A cycle threshold of 25 was chosen as a cutoff for dichotomized analysis given this was approximately the median of the group. This semi‐quantitative assay was validated by proficiency testing with the Centers for Disease Control (CDC) in which our assay performed well against the proficiency panel provided by the CDC. In addition, 35 samples were sent to the CDC during the outbreak (Figure S1). Fifteen of these 35 patients were included in the final analysis after exclusion for duplicate samples and samples collected from inpatients greater than 24 hours after admission. CT values were not available for these samples, and thus, these patients were excluded from the viral load analysis.

### 2.3 Data collection and definitions

Electronic medical records were reviewed for all pertinent demographic, clinical presentation, and outcome data. Co‐infections with viral, bacterial, or fungal pathogens were identified via laboratory reports and/or clinical examination. Suspected bacterial pneumonia was defined as an abnormal clinical and/or X‐ray findings and a decision by the medical team to treat with antibiotics.^18^, ^19^ Admission for respiratory cause was determined based on documentation indicating the primary reason for admission was for respiratory symptoms. Respiratory support was defined as any supplemental oxygen or positive pressure assistance, including but not limited to blow‐by oxygen, nasal cannula, high‐flow nasal cannula, CPAP, BIPAP, or invasive mechanical ventilation. Lower respiratory tract infection (LRTI) was defined as radiographic findings suggestive of infection or any patient requiring supplemental oxygen (confirmed) or with symptoms or examination findings consistent with LRTI (probable). A validated respiratory score based on respiratory rate, retractions, and wheeze was used as a proxy for disease severity.^20^ It is standard practice in our ED and urgent care to calculate the respiratory score on patients with symptomatic disease at the time of presentation. This respiratory score, developed at our institution, ranges from 1 to 12, with 9 to 12 indicative of severe respiratory distress, 5 to 8 of moderate distress, and 1 to 4 of mild or no respiratory distress.^20^ Respiratory score is utilized to manage patients with asthma and assist in directing therapy by healthcare personnel at our institution.

### 2.4 Statistical analysis

Patient demographic and clinical characteristics were summarized by EV‐D68 status using counts and percentages or median and range, as appropriate. Associations between confirmed EV‐D68 and clinical outcomes were explored using bivariate and multivariable logistic regression modeling. A subgroup analysis with the group of confirmed EV‐D68 patients used logistic regression modeling to investigate associations between dichotomized viral load (high: CT<25 vs. low: CT≥25) and clinical outcomes. Covariates in the multivariable models included categorical age (<1 year, 1‐5 years, and >6 years) and asthma (yes/no). All tests were two‐sided and used an alpha level of .05. Analyses were performed using Stata 12.0 (Stata Corp, College Station, TX). This study was reviewed and approved by the Institutional Review Board of Seattle Children's Hospital.

## 3 RESULTS

### 3.1 Cohort description and demographics

**Figure 1:**
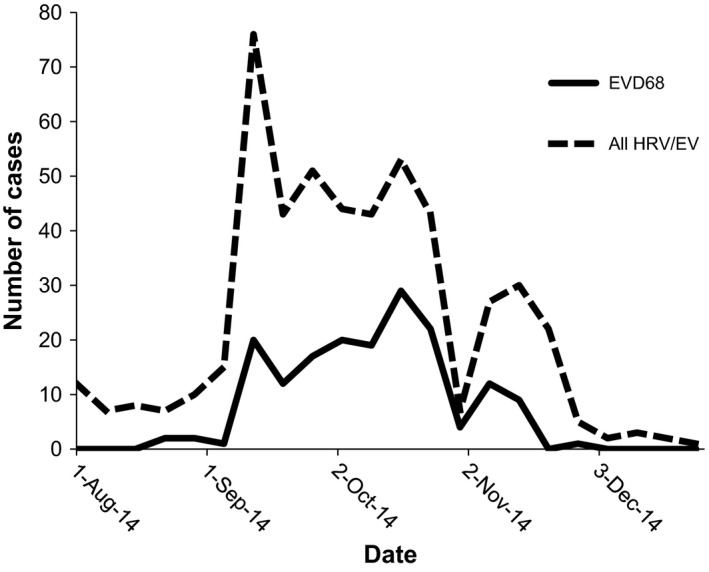
Temporal distribution of EV‐D68 and non‐D68 HRV/EV cases at Seattle Children's Hospital

Altogether, 3178 samples were tested by the FilmArray rapid PCR respiratory panel during the study period of August to December 2014. Of these, 878 (28%) samples tested positive for HRV/EV, of which 623 (71%) were available for further testing (Figure S1). Thirty‐five of these 878 samples were sent to the CDC early in the outbreak at the request of hospital and local public health authorities to ascertain the status of EV‐D68 in our state. After excluding duplicate encounters and patients tested more than 24 hours after admission, 511 patients with samples available for further testing fulfilled inclusion criteria for the study, including 15 of the 35 samples sent to the CDC. Of these 511, 170 (33%) were positive for EV‐D68 while the remaining 341 (67%) were positive for non‐EV‐D68 HRV/EV (Figure S1). The majority of patients presented between September and October 2014 (Figure 1).

Demographic data for the 511 patients tested for EV‐D68 are presented in Table 1. A slight male predominance of roughly 60% was seen in both patients with non‐EV‐D68 HRV/EV and those with EV‐D68. The median age was 3 years (range 0‐20 years) among patients with non‐EV‐D68 EV/HRV and 5 years (range 0‐17 years) among patients with EV‐D68. Subjects with EV‐D68 had higher percentages of underlying respiratory disease and asthma than those with non‐EV‐D68 EV/HRV (75% vs 52% and 68% vs 36%, respectively).

**Table 1:** Demographic characteristics

	No EV‐D68 N = 341 n (%)	EV‐D68 N = 170 n (%)
Male	202 (59)	104 (61)
Hispanic ethnicity		
Non‐Hispanic	262 (77)	125 (74)
Hispanic	67 (20)	35 (21)
Refused or unknown	12 (4)	10 (6)
Racial categories		
Caucasian	180 (53)	86 (51)
American Indian or Alaska Native	3 (1)	2 (1)
Asian	28 (8)	20 (12)
Black or African American	42 (12)	18 (11)
Native Hawaiian or Other Pacific Islander	11 (3)	2 (1)
Other	65 (19)	34 (20)
Refused or unknown	12 (4)	8 (5)
Age (y), median (range)	3 (0‐20)	5 (0‐17)
Age categories		
<1 y	84 (25)	15 (9)
1‐5 y	161 (47)	80 (47)
6+ y	96 (28)	75 (44)
Underlying disease		
Chronic immunosuppression	19 (6)	2 (1)
Hematologic malignancy	21 (6)	1 (1)
Solid tumor	13 (4)	4 (2)
Hematopoietic cell transplant	6 (2)	0 (0)
Underlying use of immunosuppressive meds	22 (6)	2 (1)
Underlying cardiac condition	25 (7)	7 (4)
Underlying respiratory	177 (52)	128 (75)
Underlying asthma	123 (36)	115 (68)
Underlying cystic fibrosis	5 (1)	1 (1)
Underlying neurologic/neuromuscular disease	29 (9)	8 (5)

### 3.2 Clinical presentation

The vast majority of patients (495/511, 97%) presented directly to the ED, with the remainder presenting to urgent care (Table 2). Five hundred and seven of 511 (99%) patients had viral swabs obtained while in ED or urgent care, while the remaining four patients were tested within 24 hours of admission. Among all patients, there was no difference between those with EV‐D68 and those with non‐EV‐D68 HRV/EV in the presence of fever or duration of symptoms. Patients with EV‐D68 had higher respiratory scores at presentation than those with non‐EV‐D68 HRV/EV (median 9 vs 6), indicating that EV‐D68‐positive patients appeared clinically more ill and had more respiratory distress at presentation. Suspected bacterial pneumonia was diagnosed in 10% in patients with EV‐D68 as compared to 3% of non‐EV‐D68 HRV/EV patients. There were very few viral co‐infections, with 6% of non‐EV‐D68 HRV/EV and 1% of EV‐D68 patients testing positive for an additional virus (Table 2). No patients were diagnosed with acute flaccid myelitis or flaccid paralysis.

**Table 2:** Clinical characteristics and disease severity

	No EV‐D68 N = 341	EV‐D68 N = 170
Location of presentation, n (%)		
Urgent Care	9 (3)	7 (4)
Emergency department	332 (97)	163 (96)
Fever, n (%)		
No	175 (51)	94 (55)
Yes	124 (36)	49 (29)
Tactile	41 (12)	27 (16)
Unknown	1 (0)	0 (0)
Days of symptoms at time of test, median (range)	2 (0‐21)	2 (0‐14)
Respiratory score at diagnosis, median (range)	6 (1‐12)	9 (1‐12)
Viral coinfection, n (%)	20 (6)	2 (1)
Bacterial coinfection, n (%)	35 (10)	20 (12)
Suspected Bacterial Pneumonia, n (%)	10 (3)	17 (10)
Fungal coinfection, n (%)	0 (0)	0 (0)

Respiratory score was available for 91% of patients with EV‐D68 (154/170) and 76% of patients with non‐EV‐D68 HRV/EV (258/341).

### 3.3 Outcomes in EV‐D68 versus non‐EV‐D68 HRV/EV

In multivariable models adjusted for the covariates of age and underlying asthma, we found that patients with EV‐D68 were more likely to require hospitalization for respiratory reasons (odds ratio: 3.11, CI: 1.85‐5.25) and to require respiratory support (odds ratio: 1.69, CI: 1.09‐2.62) (Table 3). Patients with EV‐D68 were also more likely to have severe respiratory disease involving the lower respiratory tract (confirmed LRTI odds ratio: 2.07, CI: 1.32‐3.25, confirmed or probable LRTI odds ratio: 3.78, CI: 2.03‐7.04). Furthermore, patients with EV‐D68 were more likely to require additional medical support, including the need for continuously nebulized albuterol (odds ratio: 3.91, CI: 2.22‐6.88) and initiation of steroids (odds ratio: 4.73, CI: 2.65‐8.46) (Table 3).

**Table 3:** Multivariable (adjusted) associations between clinical outcomes and EV‐D68 infection; covariates include categorical age and asthma (N = 511)

	OR (95% CI)	*P*‐value
New hospital admission, any reason	0.99 (0.58‐1.71)	.97
New hospital admission, respiratory reasons only	3.11 (1.85‐5.25)	<.01
New intensive care unit admission	0.91 (0.46‐1.79)	.79
Any respiratory support	1.69 (1.09‐2.62)	.02
Mechanical ventilation	0.38 (0.04‐3.30)	.38
Confirmed lower respiratory tract infection	2.07 (1.32‐3.25)	<.01
Confirmed or probable lower respiratory tract infection	3.78 (2.03‐7.04)	<.01
Any continuous albuterol	3.91 (2.22‐6.88)	<.01
Initiation of oral or intravenous steroids	4.73 (2.65‐8.46)	<.01

### 3.4 Association between viral load and outcomes

**Figure 2:**
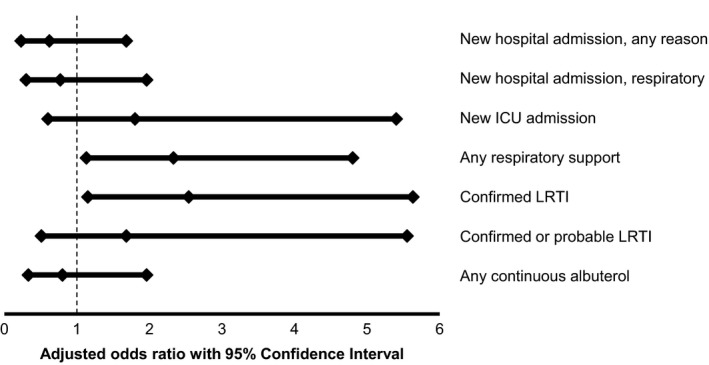
Association between clinical outcomes and Enterovirus‐D68 viral load as measured by cycle threshold. Multivariable (adjusted) associations between clinical outcomes and dichotomized EV‐D68 viral load (cycle threshold <25 vs cycle threshold ≥25). Cycle threshold ≥25 is reference. Covariates include categorical age and asthma. (N = 168)

Of 170 patients with EV‐D68 detected, 168 samples had PCR CT values reported. Multivariable logistic regression models showed evidence of increased morbidity associated with CT values <25 (higher viral load) vs. CT≥25 (lower viral load). Patients with higher viral load required respiratory support more often (OR: 2.33, CI: 1.13‐4.80) and had higher rates of confirmed LRTI (OR: 2.94, CI: 1.15‐5.63), but there was no correlation between PCR CT value and the rate of hospitalization or albuterol requirement (Figure 2).

## 4 DISCUSSION

In this report, we describe a cohort of 511 patients with EV‐D68 and non‐EV‐D68 HRV/EV who presented to the ED and urgent care at Seattle Children's Hospital during the late summer and fall of 2014. This study provides a unique focus on patients who presented to the ED or urgent care and is the largest study to date of patients with EV‐D68 in this setting.^13^, ^21^, ^22^ Our large sample size enabled us to robustly demonstrate that pediatric patients in the ED or urgent care with EV‐D68 were hospitalized, had LRTI, and required respiratory support, albuterol, and steroids more often than their counterparts with non‐EV‐D68 HRV/EV. It also enabled us to perform multivariable analyses to evaluate the effect of EV‐D68 semi‐quantitative viral load on clinical outcomes, specifically adjusting for strongly associated covariates of age and underlying asthma. Interestingly, in our study population, patients with EV‐D68 had asthma at a much higher rate than in other studies.^21^ In spite of this, when we controlled for asthma, we still found patients with EV‐D68 had higher rates of LRTI, need for continuous albuterol, and need for steroids.

The clinical severity of EV‐D68 disease in the pediatric population during the 2014 outbreak has been described by multiple groups, although primarily in hospitalized patients or special groups such as ICU patients or asthmatics.^8^, ^9^, ^10^, ^12^, ^13^, ^16^, ^22^ Of interest, nationwide surveillance for non‐influenza viral disease in the USA was limited during 2014, and so many studies have been relatively small and limited to specific geographical areas. Others have attempted to compare relative frequency of EV‐D68 during the outbreak year to other years by testing for EV‐D68 in a convenience cohort of available samples.^12^ Evaluation and sequencing of HRV/EVs in our center have been ongoing for several years.^23^, ^24^ The highest frequency of a single HRV strain was only 7% (HRV‐A10 in 2012), with most HRV types having rates of < 3% each year. These frequencies are substantially lower than the 33% incidence of EV‐D68 we detected during the 2014 outbreak.

Our study is one of the largest pediatric studies in the Western United States, and one of the few studies to exclusively characterize patients presenting to the emergency department and urgent care. We found evidence of an increased likelihood of hospitalization for respiratory symptoms in EV‐D68‐positive patients compared to patients with HRV/EV but not EVD‐68. An increased rate of hospitalization was also reported in another smaller study; however, both patients presenting to the ED and hospitalized patients were included, and thus, the absolute hospitalization rate is unclear.^22^ Our study did not find increased rates of ICU admission in unadjusted or adjusted models, possibly due to small numbers of ICU admissions in both the EV‐D68 and non‐EV‐D68 HRV/EV groups; this is consistent with several prior studies.^10^, ^13^, ^16^, ^22^

We utilized a validated respiratory score to provide a more objective characterization of clinical severity,^25^, ^26^, ^27^ although there is currently no universally adopted scoring system. The respiratory score utilized in our study was validated as a predictor for admission in asthmatic patients with good interobserver agreement.^20^, ^28^ Our study documented higher presenting respiratory scores in EV‐D68 patients, representative of a more severe clinical presentation.

Higher proportions of LRTI were seen in patients with documented EV‐D68 infection compared to non‐EV‐D68 HRV/EV; this finding remained significant after adjusting for age and underlying asthma. Other reports specifically evaluating LRTI associated with EVD‐68 have shown conflicting results, possibly due to the fact that only small numbers of patients were included and adjusted models could not be performed.^9^, ^10^ Similarly, the need for oxygen supplementation has varied among studies, which may be related in part to a lack of standardized protocols for supplemental oxygen administration, especially in circumstances of mild hypoxia, or the patient population selected for analysis.^8^, ^9^, ^12^, ^13^, ^16^, ^22^ Our data support the idea that there may be increased need for oxygen supplementation in EV‐D68‐positive patients. However, we did not see an increased need for mechanical ventilation, a finding consistent with other studies and one that is likely influenced by small sample size.^8^, ^10^, ^12^, ^13^, ^22^

Given the high rates of asthma observed in the cohort overall, we sought to understand the relationship between disease severity and asthma in the setting of EV‐D68 infection. Asthma was more common among patients with EV‐D68 than among those with non‐EV‐D68 HRV/EV. Patients with EV‐D68 were more likely to receive albuterol and steroids than their non‐EV‐D68 HRV/EV‐positive counterparts, a finding that remained significant when controlling for asthma (and age) in a multivariable analysis. Our data therefore suggest that among pediatric patients, EV‐D68 causes more severe disease than non‐EV‐D68 HRV/EV independent of asthma.

In this context, we also sought to understand the role of viral load on disease severity. Using PCR primers specific to EV‐D68, we identified that samples with PCR CT values <25 (indicating higher viral loads) were associated with worse clinical outcomes including increased need for respiratory support and increased likelihood of lower respiratory tract infections. Although other studies have demonstrated no association with viral load on similar but unique outcomes,^12^ our findings remained significant in multivariable models. This was demonstrated previously by our group for non‐EV‐D68 HRV/EV as well.^24^ In addition to qualitative detection of EV‐D68, viral load may prove useful in determining the prognosis of EV‐D68‐infected patients in the outpatient setting, although there are limitations with this assessment as cycle threshold may not be an accurate representation of viral load, and there may be variability in detection based on sample acquisition.

Our study has several additional limitations. We did not have 29% of the original 878 HRV/EV‐positive FilmArray samples available for testing, and this may have impacted the results both in the incidence of EV‐D68 and the clinical outcomes of patients with and without EV‐D68. For some subjects in our study, HRV/EV may not have been causing illness; however, nearly all subjects presented to the ED with respiratory symptoms and few had other respiratory viruses detected, suggesting that HRV/EV was playing a role.

As a tertiary care center that also serves as a community pediatric hospital for a metropolitan area, our cohort included a sample of pediatric patients likely to be representative of the greater population. However, less symptomatic patients in the community may present to primary care clinics and therefore would not be included in this study. Patients seen in the ED with minimal disease who were discharged home may not have been tested, while publicity about ongoing nationwide EV‐D68 outbreaks may have promoted higher rates of testing at our institution, with the potential for inconsistency in testing among healthcare providers. While not all ambulatory patients at our institution were captured in our study, subjects with EV‐D68 and non‐EV‐D68 HRV/EV would have been tested in the same manner because no real‐time evaluation for EV‐D68 was available at that time. Thus, comparisons between EV‐D68‐positive patients and non‐EV‐D68 HRV/EV‐positive patients should be valid assessments of disease severity. We limited our study to these two groups of patients as comparing clinical outcomes between these similar viruses could provide a basis for further research that may expose genetic explanations for the difference in disease severity. Comparing outcomes of patients with EV‐D68 to patients with other respiratory viruses would provide additional context for the burden of disease of EV‐D68 but was outside the scope of our study.

Our data suggest that among pediatric patients presenting to the ED and urgent care, EV‐D68 causes more severe disease than non‐EV‐D68 HRV/EV independent of underlying asthma and affirms that EV‐D68 was responsible for a widespread outbreak in 2014 in Seattle, WA. High viral load was associated with worse clinical outcomes, suggesting there may be a role for rapid and quantitative viral testing in order to risk stratify patients who present to the emergency department or urgent care.
