# Supplementary material for: Enterovirus D‐68 in children presenting for acute care in the hospital setting
Source: Influenza Other Respir Viruses. 2018 Mar 23;12(4):522–8. doi: 10.1111/irv.12551 (PMC6005627; doi:10.1111/irv.12551)
Supplement: Supplementary file 1 [file IRV-12-522-s001.docx]

**Supplementary Figure 1:**

878 nasal swabs positive for HRV/EV

623 available for further testing

(35 tested by CDC + 588 tested locally)

611 patient encounters after excluding duplicates

170 EV-D68 positive

(2 of these tested by CDC)

341 EV-D68 negative HRV/EV positive

(13 of these tested by CDC)

Real Time-PCR for EV-D68

511 patients presented to ED/urgent care and swabbed on presentation or within 24 hours of admission

3178 nasal swabs positive for any virus from August – December 2014
